# Making sense of the crisis: how religion shapes the attribution of meaning during the corona pandemic

**DOI:** 10.1007/s41682-022-00135-y

**Published:** 2022-11-29

**Authors:** Alexander Unser, Ulrich Riegel

**Affiliations:** 1 Faculty of Humanities and Theology, TU Dortmund University, Dortmund, Germany; 2 Department of Catholic Theology, University of Siegen, Siegen, Germany

**Keywords:** Corona pandemic, Religious meaning-making, Religiosity, Religious affiliation, RCOPE, Corona Pandemie, Religiöse Sinngebung, Religiosität, Religionszugehörigkeit, RCOPE

## Abstract

In times of existential crisis, such as the Corona pandemic, people may turn to religious traditions that help them make new sense of the depressing situation. While recent studies have shown that during the Corona pandemic, the frequency of prayer and church attendance increased in several countries, we know little about whether and how religious interpretations of the current crisis occur. Building on Crystal Park’s Meaning Making Model, the article examines whether individual religiosity, religious affiliation, and the experience of a SARS-CoV‑2 infection influence religious interpretations of the Corona pandemic. Our results show that religiosity is strongly associated with the idea of a benevolent God and weakly associated with the concept of a punishing God. Members of specific religious groups differed significantly in their religious interpretation of the Corona pandemic. Finally, we found that the experience of a SARS-CoV‑2 infection was associated with doubts about the power of God.

## Abstract (de)

In Zeiten existenzieller Krisen, wie der Corona-Pandemie, wenden sich die Menschen religiösen Traditionen zu, die ihnen helfen, der bedrückenden Situation einen neuen Sinn zu geben. Während neuere Studien gezeigt haben, dass während der Corona-Pandemie die Häufigkeiten von Gebet und Kirchenbesuchen in mehreren Ländern zugenommen haben, wissen wir bisher wenig darüber, ob und wie religiöse Interpretationen der aktuellen Krise vorgenommen werden. Aufbauend auf dem Meaning Making Model von Crystal Park wird in diesem Artikel untersucht, ob individuelle Religiosität, Religionszugehörigkeit und die Erfahrung einer SARS-CoV-2-Infektion die religiösen Interpretationen der Corona-Pandemie beeinflussen. Unsere Ergebnisse zeigen, dass Religiosität stark mit der Vorstellung eines wohlwollenden Gottes und schwach mit der Vorstellung eines strafenden Gottes verbunden ist. Weiter zeigen die Ergebnisse, dass sich Mitglieder bestimmter religiöser Gruppen in ihrer religiösen Interpretation der Corona-Pandemie signifikant unterscheiden. Schließlich zeigen die Ergebnisse, dass die Erfahrung einer SARS-CoV-2-Infektion ausschließlich mit Zweifeln an der Macht Gottes verbunden ist.

## Introduction

Global crises such as the Corona pandemic need an interpretation. It is a typically human phenomenon to search for meaning or explanation in such crisis-like events because the thought that all the experienced suffering and limitations could be due to coincidence is unbearable for many people. Coincidence and chaos deprive humans of their ability to act, while attributing meaning helps us regain at least some control and agency.

Religious traditions have always fulfilled an essential function because they provide systems of meaning that interpret the world and particular events such as pandemics (Marshall 2008; Phillips 1987). According to the existential insecurity theory (Norris and Inglehart 2004), there is even a link between the experience of one’s vulnerability and religiosity in the sense that the former has a positive effect on the latter. The theory claims that people who experience existential insecurity are, on average, more religious because their religiosity helps them to cope with the insecure situation. In modern societies of Western character, however, processes of secularisation and individualisation diminished religion’s power to orient people’s lives (Pollack 2016; Pickel 2010). According to Charles Taylor, religion may still perform as a resource of meaning but is challenged by scientific theories (Taylor 2007). Religion seems to be the least severe option to cope with life in secularised societies because of its irrational character. For example, post-truth politics outside the U.S. rely more on ethnicity and counter-science than religion (Cosentino 2020).

Given this scenario, the question arises of how religion in secular societies can still offer comfort and meaning in times of disaster. The recent SARS-CoV-2-pandemic is a master exemplar of that case. It can be understood as an individual and collective crisis affecting individual health and social and economic life. In early 2020 the world was in shock by the new virus. SARS-CoV‑2 was not yet researched well, and hardly anybody understood either the risk of contagion or the impact of this pandemic on our future life. For example, in March 2020, the German government locked down the public, cultural and economic activities in fewer than 19,000 infections and 55 deaths in a population of about 82,000,000. Although the mortality rate of SARS-CoV‑2 is higher within elderly age groups, nobody knew at this time the impact of the virus on younger people’s health. A lockdown of schools and universities resulted from this state of knowledge in early 2020. Private meetings were permitted, and many people, such as those working in bars and restaurants, lost their jobs.

In this regard, if religion still has the power to offer comfort and meaning in times of disaster (Park 2016), the SARS-CoV-2-pandemic should be the event to prove this potential. First published studies show—at least in some countries—an increase in religious practice (attendance of religious services, prayer) during the Corona pandemic (Alfano, Ercolano and Vecchione 2020; Bentzen 2021; Boguszewski et al. 2020; Molteni et al. 2020). These studies have so far focused mainly on the ritual dimension of religion. They show whether and how intensively people resort to religious rituals in the current crisis. However, whether this results in the attribution of religious meaning is not explicitly investigated. This point is not trivial for at least two reasons. First, an increase in religious-ritual practice is not necessarily an indicator that individuals interpret a situation in a religious sense. It is likewise conceivable that these rituals fulfil a social function for many people without being linked to specific religious beliefs (Marchisio and Pisati 1999; McIntosh 2015). Secondly, religious traditions offer different frameworks for interpreting crises, ranging from divine support in times of need to punishment for sin and transgression. Previous studies have shown that these interpretations can have both positive and negative effects in psychological and social terms, for example, when they influence individual well-being or susceptibility to conspiracy theories (Ano and Vasconcelles 2005; Bronstein et al. 2019). Therefore, in addition to determining whether a religious interpretation of the Corona pandemic is being made, it is crucial to understand which social conditions favour or inhibit specific religious interpretations.

In the present article, we address this research gap and investigate whether and how individual religiosity influences a religious interpretation of the Corona pandemic. Further, we examine whether belonging to a particular religious community and having experienced a SARS-CoV‑2 infection, either personally or within the family, influences the religious interpretation. For this purpose, the present study builds on Crystal Park’s (2013) Meaning Making Model, which is outlined below.

### Religion and meaning making

The Meaning Making Model deals with how people can cope with stressful life events whose demands exceed the individual’s possibilities for action. Particular attention is paid in research to events that are, in principle, uncontrollable, such as serious illnesses (cancer, HIV, etc.) or natural disasters (Park et al. 2008; Park 2016). In the face of such events, people often have no way of changing the external conditions of the situation in their favour (what is usually referred to in the literature as problem-focused coping), so they have to change the meaning of the situation to regain a certain level of comfort and well-being (Park 2013).

**Fig. 1:**
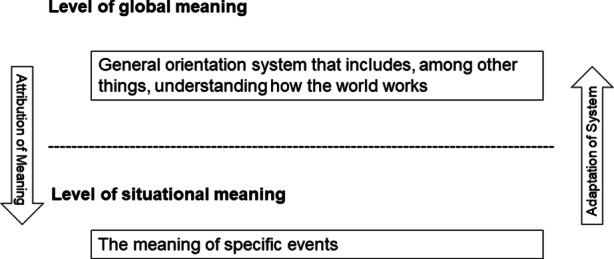
Meaning Making Process. (According to Crystal Park 2013)

The model distinguishes between two levels of meaning—the global and the situational (see Fig. 1). While the level of global meaning ‘refers to individual’s general orienting systems and view of many situations’, which includes religious beliefs, among others, the level of ‘situational meaning refers to meaning regarding a specific instance.’ (Park 2013, p. 41). The general orienting systems, located at the level of global meaning, can be considered a pool that people draw on when attributing meaning to a particular situation. Usually, people interpret certain events in the light of their general worldview; the level of global meaning influences meaning-making at the situational level. However, there can also be extreme experiences that challenge the general orienting system, so people adapt it to their situational experiences (Park 2010). Ultimately, these processes aim to ‘find a more favorable understanding of the situation and its implications’ and to ‘improve the fit between the appraised meaning of the stressor and global meaning’ (Park 2013, p. 41).

Religious traditions can play an essential role in this, as they provide beliefs, symbols and stories that can become part of the individual’s global meaning system. It is important to note, however, that religious traditions do not simply provide one specific interpretation. Instead, religious systems are characterised by being inherently plural and ambivalent. A given event can therefore be interpreted differently with recourse to the same religious tradition. Furthermore, it must be noted that religious traditions are not the only sources of global meaning systems, especially in secularised societies (Taylor 2007). It is more realistic to assume that even religious people draw on both religious and secular resources in their global meaning system so that it is not necessary that they will interpret a situation religiously.

In what follows, to reduce complexity, we will distinguish three core themes that may emerge in the religious interpretation of a crisis such as the Corona pandemic, as previous theoretical and empirical studies have shown (Padela and Curlin 2013; Pargament et al. 2000; Phillips and Stein 2007). First is the notion of a benevolent God (or higher power) who helps believers cope with loss, fear and grief or strengthens them to cope with a time of trial or a path to spiritual mastery. In the logic of this theme, crises are integrated into the wisdom of a divine plan, giving them meaning and purpose. Second is the notion of a punishing God (or higher power) who sends illness and suffering due to individual sin and wrongdoing. In this theme, disease and guilt are linked, leading the sick person to worry about their physical recovery and restoring their moral integrity. Third, a questioning of God’s power, who has not been powerful enough to help. This theme, often associated in the literature with the concepts of doubt or conversion, represents, in the logic of the Meaning Making Model, not so much an interpretation of the current situation as an adjustment of the global meaning system due to a crisis experience.

As outlined above, the ambivalent role that religion can play in meaning-making processes is very well reflected in empirical findings. In their meta-analysis of 49 studies on the impact of religious coping on psychological adjustment to stress, Ano and Vasconcelles (2005) found that positive religious interpretations of stressful life events correlated positively with self-esteem and spiritual growth and negatively with anxiety, depression or distress. Conversely, negative religious interpretations such as the concept of a punishing God were positively associated with anxiety, depression or distress. Since then, several studies have replicated these findings (Bjorck and Thurman 2007; Brelsford et al. 2015; Gerber et al. 2011).

These psychological findings are also interesting from a sociological and political point of view. Especially at the pandemic’s beginning, governments and politicians had to rely a lot on peoples’ dispositions to accept and follow the measures, cope with the additional stresses and strains, and not become addicted to conspiracy theories. Positive interpretations of the crisis are essential for this (Ano and Vasconcelles 2005; Bronstein et al. 2019). Therefore, religious traditions were possible collective resources which contributed to social cohesion at the beginning of the pandemic.

Exploring which social conditions promote or hinder such interpretations is further interesting from a sociological perspective. First, the individual experiences with a SARS-CoV‑2 infection make up such a condition. Regarding religion, there are two more possible conditions: believing and belonging. Therefore, in the following sections, we will discuss how individual religiosity as an indicator of believing, religious affiliation as an indicator of belonging and the experience of a SARS-CoV‑2 infection may impact religious meaning-making during the Corona pandemic.

### The impact of religiosity on religious meaning-making

Especially in secularised contexts such as in Western or Northern European countries (Davie 2002), there is a need to explain why people may resort to religious concepts when interpreting the Corona pandemic, as there are good reasons to believe that secular concepts are much more common there (Riegel and Unser 2021; Taylor 2007). So, under what circumstances do people resort to religious interpretations anyway?

According to the Meaning-Making model, individuals need religious resources in their global meaning system to attribute religious meaning to a particular situation. Acquiring such religious beliefs and convictions in the modern context is not a trivial issue. On the one hand, religious traditions offer relevant stories, symbols, and practices that make up religious beliefs. On the other hand, these traditions have lost much of their persuasive power. Instead, it is up to the individual to engage (or not engage) with religious traditions and to build up their global meaning system (Huber 2003; Hohenschue et al. 2022). The concept to describe the intensity of an individual’s engagement with religion is religiosity. Recent instruments often draw on Charles Glock’s (1962) conceptualisation of religiosity, which distinguishes between an ideological, intellectual, ritualistic, and experiential dimension of religion (Huber and Huber 2012; Riegel 2020). This conceptualisation has the advantage of covering religion-related beliefs, knowledge, practices and emotions, which are essential when meaning is attributed to a situation.

In line with these assumptions, several studies have found that the attribution of religious meaning is related to individual religiosity, as shown in a review by Pargament et al. (2011). This relation is, however, neither self-evident nor tautological for two reasons. First, the global meaning system of religious individuals consists for sure of religious ideas but not exclusively. It is very likely that secular explanations of the world also play an essential role in the global meaning system of religious individuals living in secularised countries. Individual religiosity is, therefore, a necessary but insufficient condition for people attributing religious meaning to a situation. Second, religious traditions are plural in themselves. They offer various religious schemes that individuals may use to interpret a particular situation. Therefore, it is necessary to research whether individual religiosity is equally related to different interpretations or whether relations are stronger with specific interpretations. Previous research, for example, has found a positive correlation between religiosity and the attribution of positive religious meaning—such as interpreting a crisis through the idea of a benevolent God (Ai et al. 2010; Freiheit et al. 2006; Lewis et al. 2005; Piderman et al. 2007; Smith et al. 2000). Accordingly, the following hypothesis is formulated.

#### Hypothesis 1a

The more religious people are, the more likely they are to interpret the Corona pandemic in the light of a benevolent God or higher power.

On the other hand, the findings on the correlation of religiosity and the attribution of negative religious meaning (such as interpreting a crisis through the idea of a punishing God) are inconsistent. While some studies could not find a significant correlation (Ai et al. 2010; Freiheit et al. 2006), other studies report a positive correlation (Lewis et al. 2005). Presumably, pastoral care in most Christian contexts abandoned the image of a punishing God. However, such ideas are still vivid in Pietist, Evangelical and Muslim environments (Boussel 2020; Cinjee and Schaap-Jonker 2021; Yendell et al. 2021). Interestingly, prominent proponents of both the Catholic and the Protestant churches in Germany felt the need to emphasise publicly that a punishing God does not issue the Corona crisis.^1^ Given these indicators, we formulate the following hypothesis.

#### Hypothesis 1b

The more religious people are, the more likely they are to interpret the Corona pandemic in the light of a punishing God or higher power.

Further, there is hardly any research on the correlation between questioning God’s power and religiosity. In a secular environment questioning God’s power should be the default mode of meaning-making. The exception of this default mode is highly religious individuals who count on God’s presence even these days. It is precisely the character of a high centrality of religion to trust in God even in times of disaster (Huber 2003). Therefore, the following hypothesis is formulated.

#### Hypothesis 1c

The less religious people are, the more likely they are to question God’s power in the face of the Corona pandemic.

### The impact of religious affiliation on religious meaning-making

As previously seen, individual religiosity cannot be conceptualised independently from religious traditions. These traditions coin the cultural environment in which religiosity and spirituality are developed and lived. Perhaps there might have been some spiritual revolution which established the personal self as the focus of one’s well-being (Heelas and Woodhead 2007). But even if the concept of spirituality became more relevant in the last decades, it did not replace the cultural sediments of traditional religious narratives, symbols, and practices. For example, as previously elaborated, the images of both a benevolent God and a punishing one still seem to be vivid. From a theoretical perspective, religious traditions represent particular conceptions of some transcendent power. But even within the so-called Abrahamic traditions, the understanding of God and devout life differ distinctively (Woodhead et al. 2016). This indicates that religious affiliation might impact religious meaning-making in times of disaster. For example, during the first year of the Corona pandemic, predominantly minority religious groups like Pentecostal and charismatic-oriented congregations were perceived as preaching a God who punishes people for their sins (Yendell et al. 2021, p. 50–53). Being affiliated with some religious community might impact meaning-making.

However, the aspect of religious affiliation hardly plays a role in research on religious meaning-making. For example, in the studies summarised by Pargament et al. (2011), several samples consist of members of different religious groups. Whether there are significant differences in positive or negative religious meaning-making is not reported in any of them. In contrast to the assumption that members of religious minority groups prefer negative religious meaning-making, Abu-Raiya and Pargament (2015) report in their literature review that participants of studies with non-Christian samples—as those of studies with Christian samples—show positive religious meaning-making more frequently than negative religious meaning-making. A difference between religious groups was only found in the extent to which religious meaning-making is used. According to Adam and Ward (2016), members of religious minority groups show a higher degree of religious meaning-making than members of majority groups. Whether members of religious minority or majority groups doubt the power of God more often in crises has not been researched yet.

In summary, there is some evidence that being a member of a religious minority may indicate a higher degree of religious coping. If so, this coping can refer to both benevolent and punishing God. In contrast, being a member of a religious majority may result in more questioning of God’s power if compared with members of religious minorities. Finally, there is no evidence whether this effect of religious affiliation is direct or moderating. This brings about two sets of hypotheses to be tested. The first set is about a possible direct effect of religious affiliation.

#### Hypothesis 2a

Members of religious minorities are more likely to interpret the Corona pandemic in the light of a benevolent God or higher power than members of a majority religion.

#### Hypothesis 2b

Members of religious minorities are more likely to interpret the Corona pandemic in the light of a punishing God or higher power than members of a majority religion.

#### Hypothesis 2c

Members of religious minorities are less likely to question the power of God in the face of the Corona pandemic compared to members of a majority religion.

The second set of hypotheses is about a moderating effect of religious affiliation since religious belonging might predict the degree of religiosity. Such moderating effects of religious affiliation have already been demonstrated in other research areas in the sociology of religion (Unser and Ziebertz 2020; Ziebertz and Unser 2020). However, such an investigation is still pending in religious meaning-making.

#### Hypothesis 3a

Belonging to a religious minority increases the positive effect of religiosity on an interpretation of the Corona pandemic in the light of a benevolent God or higher power.

#### Hypothesis 3b

Belonging to a religious minority increases the positive effect of religiosity on an interpretation of the Corona pandemic in the light of a punishing God or higher power.

#### Hypothesis 3c

Belonging to a religious minority diminishes the effect of religiosity on questioning God’s power in the face of the Corona pandemic.

### The impact of SARS-CoV-2 infection on religious meaning-making

Experiencing an infection with SARS-CoV‑2, personally or within the family, changes many things, presumably also the interpretation of the Corona pandemic. As a result, an abstract danger suddenly becomes an existential health threat. This is even more true at the beginning of the pandemic when its consequences could not have been estimated due to missing knowledge about that virus. We, therefore, assume that the experience of an infection moderates the influence of religiosity on religious meaning-making. Such an assumption can be based, among others, on the existential insecurity theory. According to Norris and Inglehart (2004), individual religiosity increases due to experiences of one’s own vulnerability to cope with it. In line with this theory, Molteni et al. (2020) have shown that the frequency of attendance at religious services and private prayers generally increased in Italy during the Corona pandemic, but that at the same time, there were significant differences between those respondents who reported SARS-CoV‑2 infection in the family and those who did not. The former showed higher frequencies compared to the latter.

While this indicates that the experience of a SARS-CoV‑2 infection stimulates recourse to religious rituals, we know little about how this affects the interpretation of the Corona pandemic. Previous studies indicate a link between strongly negative experiences and negative religious meaning-making. Bjorck and Thurman (2007), for example, have shown that in the face of an increase in negative life events, people reappraise their view of God and tend toward negative religious meaning-making. Ai et al. (2007) have also demonstrated the influence of crisis experiences on negative religious meaning-making. Still, based on their findings that negative religious meaning-making is not linked to religiosity, they assume that ‘[s]uch styles, although with faith content, may have more to do with distress than with strength of faith’ (Ai et al. 2007, p. 878). Against the research literature’s background, we formulate the following hypotheses.

#### Hypothesis 4a

The experience of infection with SARS-CoV‑2 has no influence on the interpretation of the Corona pandemic in the light of a benevolent God or higher power.

#### Hypothesis 4b

The experience of infection with SARS-CoV‑2 has a direct positive influence on the interpretation of the Corona pandemic in the light of a punishing God or higher power but does not moderate the impact of religiosity on this interpretation.

#### Hypothesis 4c

The experience of infection with SARS-CoV‑2 does not influence whether the power of God is questioned in the face of the Corona pandemic.

## Data and method

The present study’s data come from a questionnaire on how students and university staff cope with the Corona pandemic, distributed at two German universities (both in the federal state of North Rhine-Westphalia) in April 2020.

At this time, the Corona pandemic led to a strict lockdown of public life in March 2020 in Germany. On 03/17, non-citizens were not allowed to enter Germany anymore for private reasons, and shops that did not sell goods for daily necessities were closed. On 03/22, people were asked to stay home and avoid contact with others. At universities, staff had to work from home, affecting the type of communication within the universities’ administration and most office routines. Consequently, university staff had to cope with the social lockdown and the changes in the daily workflow. Students were not allowed to enter university too. That meant online lectures, no library service, no access to laboratories, no student life on campus, etc. Many lost their jobs during that time because typical student jobs, such as waiters in bars and restaurants, were terminated. The strict lockdown of public life was in effect until mid of April 2020. Because the infection rate started to decrease then, the governmental bodies agreed to re-open public life step by step on 04/15. From 04/20, people were allowed to leave home for sports or to take a stroll if they did not meet with others, and small shops were allowed to open again. But still, strict sanitation measures had to be obeyed, like keeping a distance of at least 1.5 m to others, wearing a face mask in public, etc. The situation at universities was not affected by these measures. Students and staff still had to cope with the fact that hardly anybody could enter university facilities and use university services other than virtual ones. And there still was barely any knowledge on the virus and how the infection will affect the individual’s life.

The questionnaire was accessible between 04/16 and 04/26 when the strict lockdown was still in effect, but Germany started to discuss the first measures to soften it. Students and staff were informed about the study via the faculties’ mailing lists at the two universities where the data was collected. That mail informed about the inquiry’s goal and reassured the privacy of the endeavour and that nobody would face any problem if one did not participate in the study. It also provides students and staff with the link to the questionnaire.

Overall, N = 2,670 respondents filled in the questionnaire. Most of the sample is female (72.0%), born in 1995 or later (56%). Only 236 participants are older than 41 years. The latter reflects that 2,151 (81%) students participated in the study and 519 (19%) staff members. This composition already shows that the study sample does not represent the population of Germany in general, for example, regarding the age structure and the level of formal education. This methodological limitation is reflected in more detail below in the Discussions part.

A variable assessed the faculties scientific background a student is enrolled in or a staff member is working in. According to this variable, 715 (27%) respondents are associated with social sciences, 699 (26%) with humanities, 500 (19%) with mathematics or natural sciences, and 341 (13%) with economy. The rest did not fill in this variable for various reasons (15%).

In terms of religious affiliation, 518 participants (20.4%) indicated that they were non-denominational, 937 (36.9%) that they were Roman Catholic, 755 (29.7%) that they were Protestant, 117 (4.6%) that they belong to a Free Christian Church (which includes, for example, Baptist churches and Pentecostal churches), 154 (6.1%) that they were Muslim, and 61 (2.3%) that they belonged to another religious tradition (such as Christian Orthodoxy, Judaism, Buddhism and Hinduism). The rest of the sample did not fill this category and were therefore excluded from the analyses.

### Dependent variables

The dependent variables of the present study measure whether and how respondents ascribe religious meaning to the Corona pandemic. We focus here on the three core themes outlined above that may emerge in the religious interpretation of a crisis: the notion of a benevolent God (or higher power), the idea of a punishing God (or higher power) and the questioning of God’s power. These three core themes were operationalised through three subscales (namely the Benevolent God Reappraisal subscale, the Punishing God Reappraisal subscale and the Reappraisal of God’s Power subscale) of the RCOPE scale (Pargament et al. 2000), a widely used instrument for researching religious coping (the wording of the items is documented in Table 5 in the appendix). In our study, we used the three-item versions of the respective subscales. The three subscales showed acceptable to good internal consistencies. The Benevolent God Reappraisal subscale reached a Cronbach’s-alpha of 0.87, the Punishing God Reappraisal subscale a Cronbach’s-alpha of 0.76, and the Reappraisal of God’s Power subscale a Cronbach’s-alpha of 0.66.

Table 1 shows that religious interpretations of the Corona pandemic are not widespread in the sample, reflecting the secularised context in Germany well. On a 5-point scale ranging from −2 to 2, the Benevolent God Reappraisal subscale only achieves a mean value of −1.10, the Reappraisal of God’s Power subscale a mean value of −1.49 and the Punishing God Reappraisal subscale even only a mean value of −1.82. The very low standard deviation (SD) should also be noted in the latter’s case.

**Table 1:** Descriptive Statistics

	Min	Max	Mean	SD	Perc
*Religious Reappraisal of the Pandemic*					
Benevolent God Reappraisal	−2	2	−1.10	1.12	–
Punishing God Reappraisal	−2	2	−1.82	0.45	–
Reappraisal of God’s Power	−2	2	−1.49	0.81	–
Religiosity	−2	2	−0.43	1.13	–
*Affiliation*					
Catholic	0	1	–	–	36.9
Protestant	0	1	–	–	29.7
Christian Free Church	0	1	–	–	4.6
Muslim	0	1	–	–	6.1
Other religious tradition	0	1	–	–	2.3
Non-affiliated	0	1	–	–	20.4
*SARS-CoV‑2 infection*	0	1	–	–	15.2
*Sex*					
Female	0	1	–	–	72.0
Male	0	1	–	–	28.0
*Status*					
Student	0	1	–	–	81.0
Staff	0	1	–	–	19.0

### Independent variables

In our analyses, we examine the influence of three independent variables on religious interpretations of the Corona pandemic: religiosity, religious affiliation, and the experience of infection with SARS-CoV‑2.

Religiosity is operationalised in this study through the five-item version of the Centrality of Religiosity Scale (CRS) (Huber and Huber 2012). This scale measures religiosity as a multidimensional construct that includes the intellectual dimension, the ideological dimension, the dimensions of public and private practice, and the experiential dimension (the wording of the items is documented in Table 5 in the appendix). Following Huber and Huber’s (2012, p. 720) instructions, the two items measuring the public and private practice were recoded so that all five items have a range of values between 1 and 5. The scale shows good internal consistency with a Cronbach’s‑α value of 0.90. Due to the moderator analyses carried out in the following, the value three was subtracted from the CRS so that the value range now lies between −2 and 2 (see Table 1).

Religious affiliation is measured by an item that asks respondents to self-report their affiliation. The distribution of the different religious traditions has already been outlined above in the sample description. In addition, we introduced the distinction between majority and minority religious groups in the theoretical part of the article, which now needs to be clarified here concerning the sample. In Germany, there are two large denominational groups: the Catholics and the Protestants. The latter are incorporated in the Evangelical Church in Germany (EKD). Both Catholics and Protestants represent about 25% of the population. These two denominations represent religious majority groups in Germany. In this study, however, we also include the religiously non-affiliated, who comprise more than a third of the German population. The religious minority groups counted in this study are the Muslims, members of Christian Free Churches and of other religious traditions, each of which is estimated to make up less than 5% of the German population.

Experience of infection with SARS-CoV‑2, either personally or within the family, was measured by three items that asked respondents whether they (item 1), their partner or children (item 2) or other relatives such as parents, siblings, uncles or aunts (item 3) had tested positive for SARS-CoV‑2. If respondents indicated yes to at least one item, they were assigned a value of 1. If they stated no for all three items, they were given a value of 0. Table 1 shows that about 15.2% of the respondents had experienced a SARS-CoV‑2 infection when completing the questionnaire.

In addition to these three independent variables, we also included the sex and status of the respondents as control variables. The age of the respondents showed no effect, which is why we excluded this variable from further analyses.

### Statistical analyses

To test the hypotheses formulated above, we conducted OLS-regression analyses. Interaction terms were computed between religiosity on the one hand and religious affiliation or infection with SARS-CoV‑2 on the other to test the moderator hypotheses. All analyses were carried out with version 28 of the statistical program SPSS.

## Results

The following sections present the results of the analyses. In the first step, the influences of all independent and control variables on the three dependent variables are estimated using OLS regression models (Sect. 3.1). Then, interaction effects between religious affiliation (Sect. 3.2) and the experience of a SARS-CoV‑2 infection (Sect. 3.3) on the one hand and religiosity, on the other hand, are computed to test the moderation hypotheses.

### Predictors for the attribution of religious meaning to the Corona pandemic

Table 2 shows the results of the regression analyses with the subscale Benevolent God Reappraisal as the dependent variable. The control variables and the three independent variables are entered into the model stepwise so that the increase in R^2^ indicates the contribution of each variable to the explanatory power of the model.

**Table 2:** The Influence of Religiosity, Religious Affiliation, and Infection on the Benevolent God Reappraisal of the Corona Pandemic

	Model 1	Model 2	Model 3	Model 4
	Coeff. (s.e.)	Coeff. (s.e.)	Coeff. (s.e.)	Coeff. (s.e.)
Intercept	−1.51* (0.06)	−0.98* (0.04)	−1.10* (0.04)	−1.09* (0.04)
*Sex (ref.* *=* *Male)*				
Female	0.26* (0.05)	0.07* (0.03)	0.07* (0.03)	0.08* (0.03)
*Status (ref.* *=* *Staff)*				
Student	0.26* (0.06)	0.17* (0.04)	0.15* (0.04)	0.15* (0.04)
Religiosity	–	0.78* (0.01)	0.73* (0.02)	0.73* (0.02)
*Affiliation (ref.* *=* *Catholic)*				
Protestant	–	–	0.06 (0.03)	0.06 (0.03)
Christian Free Church	–	–	0.43* (0.07)	0.43* (0.07)
Muslim	–	–	0.76* (0.07)	0.76* (0.07)
Other religious tradition	–	–	0.36* (0.10)	0.36* (0.10)
Non-affiliated	–	–	0.15* (0.04)	0.15* (0.04)
*SARS-CoV‑2 infection*	–	–	–	−0.03 (0.04)
*Sample Size*	2338	2337	2332	2331
*Adj. R*^*2*^	0.02	0.63	0.65	0.65

*p < 0.05

As can be seen, R^2^ increases most strongly from model 1 to model 2 when religiosity is entered as a predictor (∆R^2^ = 0.61). This reflects quite well the high predictive power of religiosity, which is strong (b-value = 0.73 in model 4). In contrast, all other predictors slightly increase the model’s explanatory power (max ∆R^2^ = 0.02).

The results in model 4 also show that religiosity positively influences a religious interpretation of the Corona pandemic, which refers to a benevolent God (confirmation of hypothesis 1a). Furthermore, significant differences can be found between religious minority groups (b-value of Christian Free Churches = 0.43; b‑value of Muslims = 0.76; b‑value of other religious traditions = 0.36; all compared to Catholic respondents) on the one hand and religious majority groups (no significant differences between Catholic and Protestant respondents) on the other hand to the effect that the former have higher scores in interpreting the Corona pandemic in the light of a benevolent God compared to the latter (confirmation of hypothesis 2a). Surprisingly, even the non-affiliated respondents differ significantly from the Catholic respondents and show higher scores in the Benevolent God Reappraisal subscale. As expected, the experience of a SARS-CoV‑2 infection has no significant effect in this model (confirmation of hypothesis 4a).

The results of the regression analyses with the subscale Punishing God Reappraisal as the dependent variable are documented in Table 3. First of all, it should be noted that this model’s explanatory power is considerably lower than the previous one (R^2^ = 0.14). Including the independent variables religiosity (∆R^2^ = 0.08) and religious affiliation (∆R^2^ = 0.05) increases the explanatory power to a comparable extent in each case.

**Table 3:** The Influence of Religiosity, Religious Affiliation, and Infection on the Punishing God Reappraisal of the Corona Pandemic

	Model 1	Model 2	Model 3	Model 4
	Coeff. (s.e.)	Coeff. (s.e.)	Coeff. (s.e.)	Coeff. (s.e.)
Intercept	−1.92* (0.02)	−1.84* (0.02)	−1.83* (0.03)	−1.83* (0.03)
*Sex (ref.* *=* *Male)*				
Female	0.01 (0.02)	−0.01 (0.02)	−0.02 (0.02)	−0.02 (0.02)
*Status (ref.* *=* *Staff)*				
Student	0.09* (0.02)	0.08* (0.02)	0.06* (0.02)	0.06* (0.02)
Religiosity	–	0.11* (0.01)	0.10* (0.01)	0.10* (0.01)
*Affiliation (ref.* *=* *Catholic)*				
Protestant	–	–	−0.02 (0.02)	−0.02 (0.02)
Christian Free Church	–	–	−0.17* (0.04)	−0.17* (0.04)
Muslim	–	–	0.36* (0.04)	0.36* (0.04)
Other religious tradition	–	–	0.21* (0.06)	0.20* (0.06)
Non-affiliated	–	–	−0.01 (0.03)	−0.01 (0.03)
*SARS-CoV‑2 infection*	–	–	–	−0.02 (0.02)
*Sample Size*	2338	2337	2332	2331
*Adj. R*^*2*^	0.01	0.09	0.14	0.14

*p < 0.05

The regression coefficients show that religiosity positively influences (b-value = 0.10 in model 4) a religious interpretation of the Corona pandemic that refers to a punishing God (confirmation of hypothesis 1b). It should be noted, however, that this influence is minimal. Even when the variable religiosity reaches the highest value, this does not yet lead to an agreement with the interpretation of the Corona pandemic as a punishment by God in the sample, but instead to a less strong rejection of this interpretation. The same applies to the differences in religious affiliation. Both members of Christian free churches and other religious traditions, as well as Muslims, differ significantly from Catholic respondents, while there are no significant differences between Catholics and Protestants. However, while Muslims (b-value = 0.36) and members of other religious traditions (b-value = 0.20) are less opposed to the interpretation of the Corona pandemic as God’s punishment, members of Christian Free Churches (b-value = −0.17) are even more opposed to this interpretation than Catholics. In this case, there is no simple juxtaposition of religious majorities and religious minorities; instead, the situation is more complex (partial confirmation of hypothesis 2b). Furthermore, contrary to expectations, the experience of a SARS-CoV‑2 infection has no significant effect (rejection of hypothesis 4b).

Finally, Table 4 shows the results of the regression analyses with the subscale Reappraisal of God’s Power as the dependent variable. The very low R^2^ of 0.02 indicates that the model’s independent variables hardly contribute to explaining differences in the dependent variable.

**Table 4:** The Influence of Religiosity, Religious Affiliation, and Infection on the Reappraisal of God’s Power during the Corona Pandemic

	Model 1	Model 2	Model 3	Model 4
	Coeff. (s.e.)	Coeff. (s.e.)	Coeff. (s.e.)	Coeff. (s.e.)
Intercept	−1.59* (0.04)	−1.57* (0.05)	−1.47* (0.05)	−1.48* (0.05)
*Sex (ref.* *=* *Male)*				
Female	−0.08* (0.04)	−0.09* (0.04)	−0.09* (0.04)	−0.10* (0.04)
*Status (ref.* *=* *Staff)*				
Student	0.18* (0.04)	0.18* (0.04)	0.17* (0.04)	0.17* (0.04)
Religiosity	–	0.03* (0.02)	0.05* (0.02)	0.05* (0.02)
*Affiliation (ref.* *=* *Catholic)*				
Protestant	–	–	−0.09* (0.04)	−0.09* (0.04)
Christian Free Church	–	–	−0.31* (0.09)	−0.31* (0.09)
Muslim	–	–	−0.29* (0.08)	−0.29* (0.08)
Other religious tradition	–	–	−0.07 (0.11)	−0.06 (0.11)
Non-affiliated	–	–	−0.14* (0.05)	−0.13* (0.05)
*SARS-CoV‑2 infection*	–	–	–	0.10* (0.02)
*Sample Size*	2338	2337	2332	2331
*Adj. R*^*2*^	0.01	0.01	0.02	0.02

*p < 0.05

Nevertheless, a small positive influence of religiosity (b-value = 0.05 in model 4) on the reappraisal of God’s power in the face of the Corona pandemic can be observed (rejection of hypothesis 1c), as well as an unexpected, small positive influence of an experience with a SARS-CoV‑2 infection (b-value = 0.10; rejection of hypothesis 4c), although both effects are probably below the limits of practical relevance. The differences between religious groups do not follow the boundaries of majority and minority groups this time. Rather, members of all religious groups—except members of other religious traditions—are less likely to doubt the power of God in the face of the Corona Pandemic compared to Catholics. This applies to Protestants (b-value = −0.09), members of Christian Free Churches (b-value = −0.31), Muslims (b-value = −0.29) and the non-affiliated (b-value = −0.13; partial confirmation of hypothesis 2c).

### Interaction effects between religious affiliation and religiosity

**Fig. 2:**
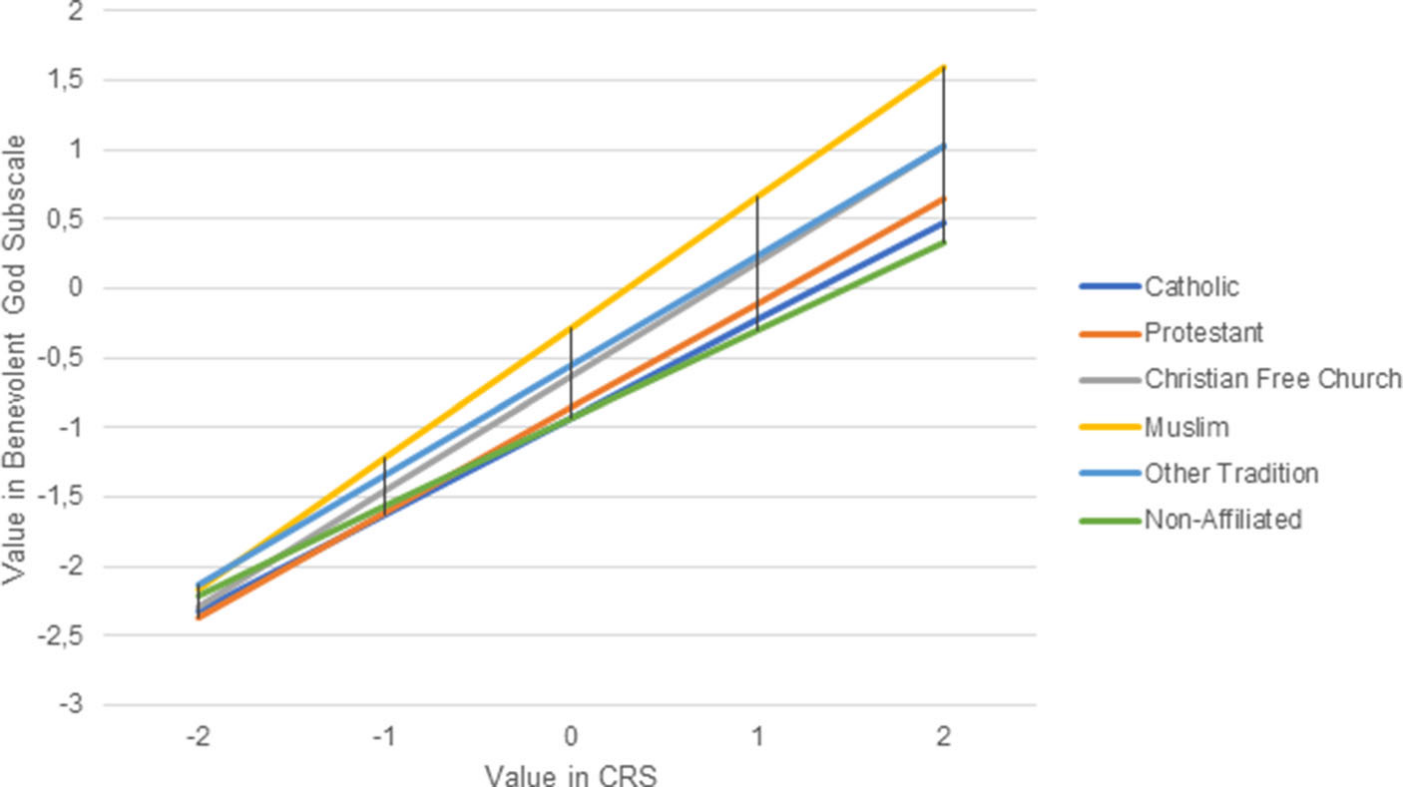
Religious affiliation moderating the influence of religiosity on the Benevolent God Reappraisal of the Corona Pandemic (The influence of the religiosity of Muslims (b-value = 0.94) differs significantly from the influence of the religiosity of Catholics (b-value = 0.70; *p* < 0.001), while the differences between Catholics and members of all other religious groups are not significant (*p* > 0.05))

In a second step, the results of the moderation analyses will be presented, in which it will be examined whether religiosity has a different impact among individual religious groups. For this purpose, the different regression slopes are presented graphically and discussed. Fig. 2 shows the regression slopes of religiosity (with the dependent variable Benevolent God Reappraisal) for the respective religious groups. Once again, Catholics (dark blue line) are the reference category.

Graphically, it can be seen that the slopes (b-values) of the individual regression lines differ from each other. On the one hand, the non-affiliated, the Catholics and the Protestants, and the Christian Free Churches and other religious traditions, on the other, show similar slopes. Muslims are set apart from the rest. In a statistical sense, however, only the difference between Muslims (b-value = 0.94) and Catholics (b-value = 0.70) can be shown to be significant (*p* < 0.001). Thus, religiosity has a stronger influence among Muslims than Catholics on an interpretation of the Corona Pandemic in the light of a benevolent God. Hypothesis 3a, which assumes a fundamental difference between religious minorities and majorities, must be rejected.

**Fig. 3:**
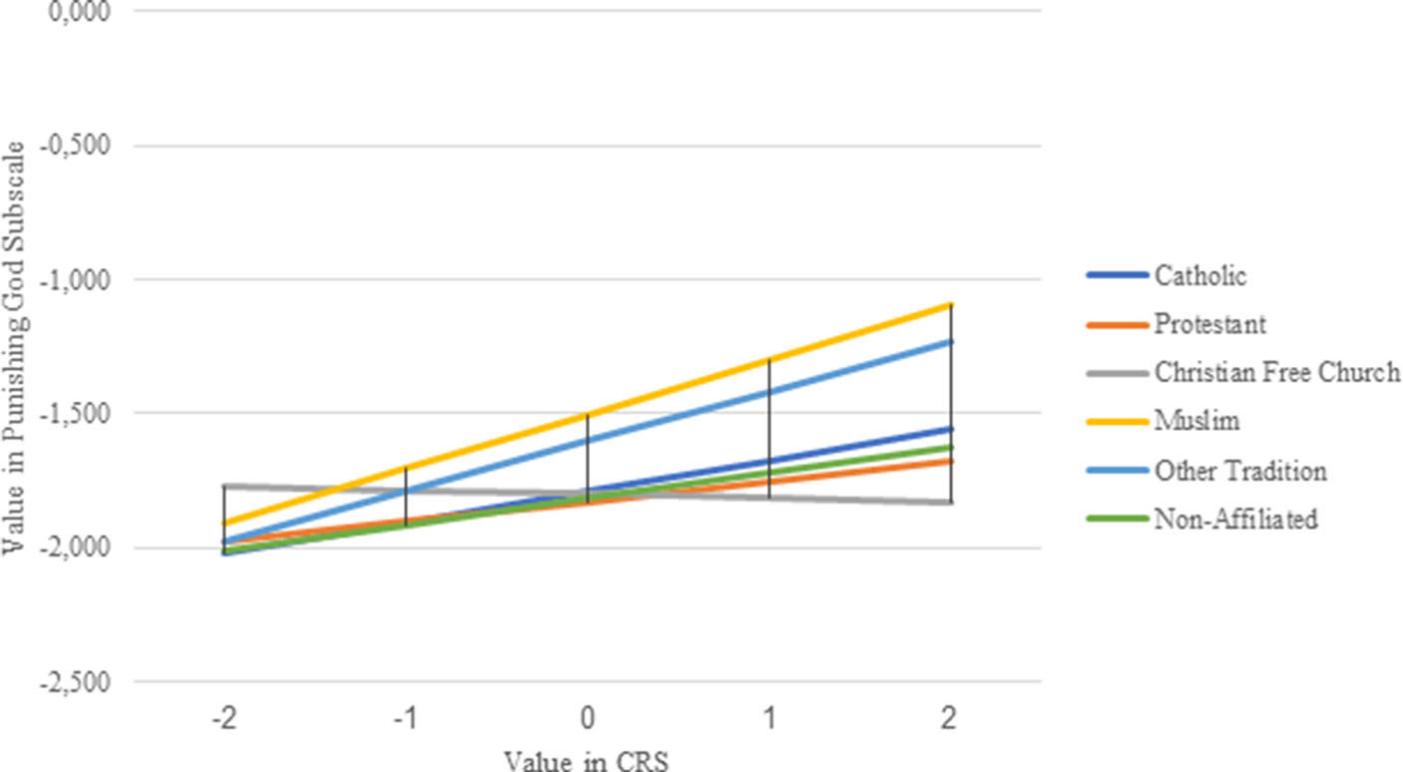
Religious affiliation moderating the influence of religiosity on the Punishing God Reappraisal of the Corona Pandemic (The influence of the religiosity of Catholics (b-value = 0.12) differs significantly from the influence of the religiosity of Muslims (b-value = 0.20; *p* = 0.039) and members of Christian Free Churches (b-value = −0.02; *p* = 0.005), while the differences between Catholics and members of all other religious groups are not significant (*p* > 0.05))

Fig. 3 shows the regression slopes of religiosity (with the dependent variable Punishing God Reappraisal) for the respective religious groups with Catholics (dark blue line) as the reference category. Here, too, similar slopes can be found among the religious groups. While the Muslims and the other religious traditions show the highest slopes, the Catholics, the non-affiliated and the Protestants are set apart. On the other hand, the Christian Free Churches are the only religious group that shows a negative slope.

Here, too, only a few differences can be identified as significant in a statistical sense. It is found that the religiosity of Muslims (b-value = 0.20), compared to the religiosity of Catholics (b-value = 0.12), has a significantly stronger influence on an interpretation of the Corona pandemic as God’s punishment (*p* = 0.039). Furthermore, the influence of the religiosity of the Christian Free Churches, which is close to 0 (b-value = −0.02), differs from the influence of the religiosity of Catholics (*p* = 0.005). Again, a simple distinction between religious minorities and religious majorities does not work, which is why hypothesis 3b is rejected.

**Fig. 4:**
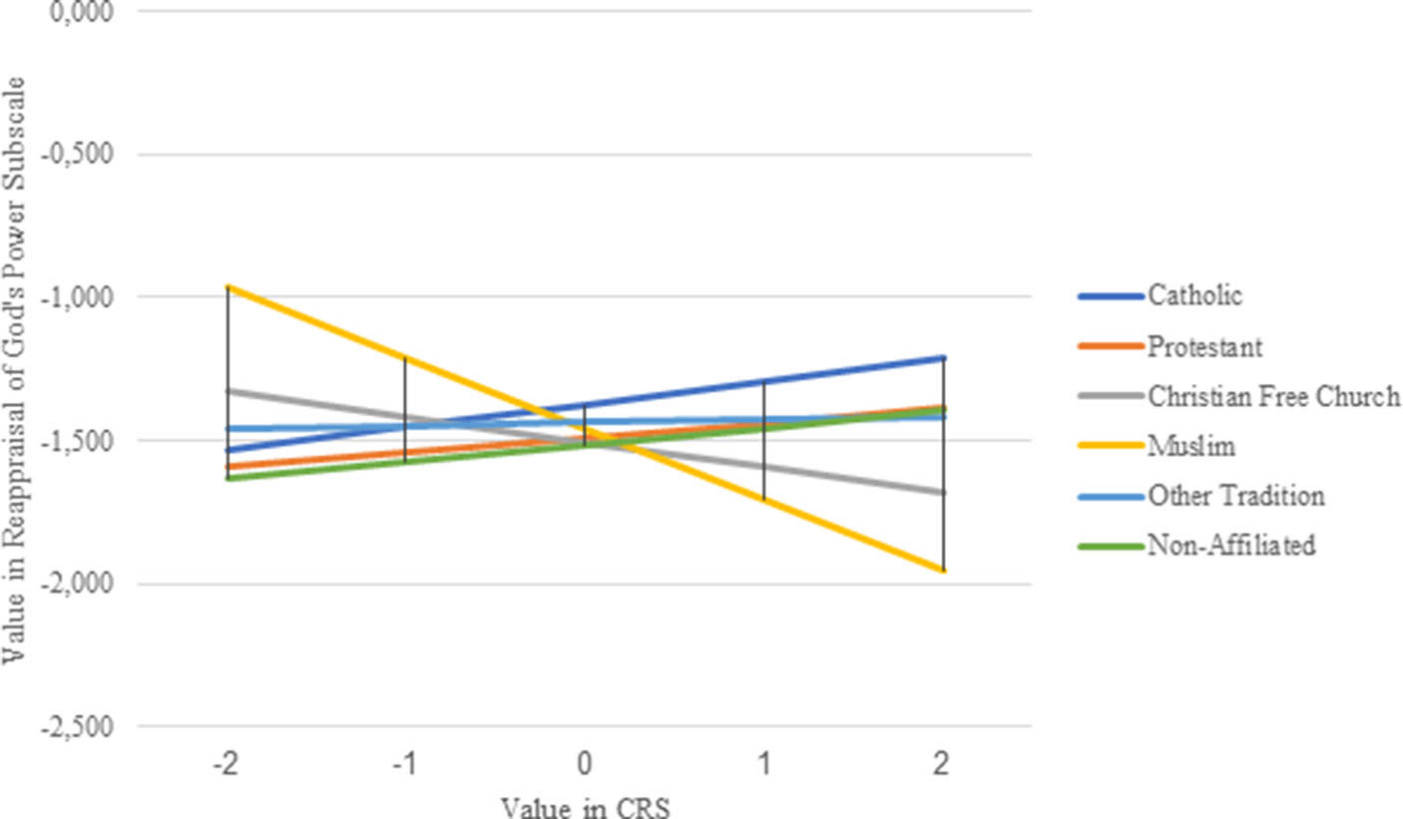
Religious affiliation moderating the influence of religiosity on the Reappraisal of God’s Power during the Corona Pandemic (The influence of the religiosity of Catholics (b-value = 0.08) differs significantly from the influence of the religiosity of Muslims (b-value = −0.25; *p* < 0.001), while the differences between Catholics and members of all other religious groups are not significant (*p* > 0.05))

Finally, Fig. 4 shows the regression slopes of religiosity (with the dependent variable Reappraisal of God’s Power) for the respective religious groups, with Catholics (dark blue line) as the reference category. Here, slightly positive slopes can be seen among Catholics, Protestants and the non-denominational, while the slope for the other religious traditions is around 0 and the slopes for Christian Free Churches and Muslims are negative.

However, only the differences between Catholics (b-value = 0.08) and Muslims (b-value = −0.25) are significant in a statistical sense (*p* < 0.001). Religiosity has a different impact on the two religious groups, not only in terms of strength but also in the direction of influence. While among Catholics, religiosity slightly increases doubt in God’s power during the Corona pandemic, religiosity tends to prevent doubt among Muslims. Accordingly, hypothesis 3c, which assumes that religious minorities do not question God’s power in times of disaster like religious majorities, can partly be confirmed.

### Interaction effects between the experience of a SARS-CoV-2 infection and religiosity

The final step is to discuss the results of the moderation analyses, which examined whether religiosity has a different influence when respondents have experienced SARS-CoV‑2 infection. The three analyses with Benevolent God Reappraisal, Punishing God Reappraisal, and Reappraisal of God’s Power as dependent variables showed no significant interaction between religiosity and infection (*p* > 0.05). Thus, the existential crisis of a SARS-CoV‑2 infection does not lead to a stronger or weaker influence of religiosity, as expected for the Benevolent God Reappraisal and the Reappraisal of God’s Power, but not for the Punishing God Reappraisal (rejection of hypothesis 4b).

## Discussion

This paper aimed to investigate whether and how people ascribe religious meaning to the Corona pandemic and whether their religiosity, religious affiliation and experience of SARS-CoV‑2 infection influence this. Given the recent sociological discussion on secularisation (Pollack 2016; Pollack and Pickel 2007; Taylor 2007), this question is of particular relevance because it is not clear to what extent religion unfolds its meaning-giving power under the present conditions. Religious meaning-making is associated with mental health (Ano and Vasconcelles 2005) and susceptibility to fake news and conspiracy theories (Bronstein et al. 2019). Both aspects are relevant for sociological and political research on the Corona pandemic because they point at religion and religious-meaning making as a resource of social cohesion, especially in terms of obeying and enduring the measures imposed by the government. There have mainly been studies on the increase in religious practice during the Corona pandemic (Alfano et al. 2020; Bentzen 2021; Boguszewski et al. 2020; Molteni et al. 2020), so our article fills a significant research gap.

Our findings confirm several previous studies but also show that further investigations are necessary about differences between religious groups. In line with several previous studies (Ai et al. 2010; Freiheit et al. 2006; Lewis et al. 2005; Pargament et al. 2011; Piderman et al. 2007; Smith et al. 2000), religiosity was found to influence religious meaning-making. Likewise, our findings revealed a tendency that has already become clear in previous studies. There is a stronger influence of religiosity on positive religious meaning-making (in our study Benevolent Religious Reappraisal) compared to the impact of religiosity on negative religious meaning-making (in our study Punishing God Reappraisal). On the other hand, religiosity’s influence on questioning God’s power was very low. These findings show that during the Corona pandemic, people draw on their religious resources to cope with the crisis just as they do, for example, in the face of natural disasters or deadly diseases such as cancer or HIV. This is even more relevant since our sample consists predominantly of young students representing an age cohort which has mainly been socialised in a secularising cultural environment. At the beginning of the Corona pandemic, when the consequences of the virus had not been understood exactly, even many of these youngsters turned to some extent to religion to cope with the pandemic. In this context, religion as a resource is activated to a much greater extent to make a positive interpretation of the Corona pandemic, compared to negative interpretations, which, at least in our sample, hardly receive any support even among religious respondents. This tendency runs through all religious groups, whether religious majorities or minorities. Accordingly, a mainly positive impact of religion on dealing with the measures and on social cohesion can be expected, although we did not research this effect.

Nevertheless, our findings show significant differences in the attribution of religious meaning between members of different religious traditions. To our knowledge, no studies have conducted comparative analyses of this, which means that our article fills an important research gap (Abu-Raiya and Pargament 2015). In formulating our hypotheses, we were guided by the assumption of Adam and Ward (2016). They claim that members of religious minority groups use religious meaning-making to a greater extent than members of religious majority groups. Such a distinction between minority and majority religious groups has proven to be a valid first approximation to the phenomenon in our analyses. Members of religious minority groups (Christian Free Churches, Muslims, and other religious traditions) showed significantly higher scores on Benevolent Religious Reappraisal and, in some cases, significantly higher scores on Punishing God Reappraisal than members of religious majority groups.

Further, Muslims and Christian Free Churches members showed significantly lower scores on the Reappraisal of God’s Power. Accordingly, a religious community’s social position moderates religion’s effect in coping with disaster. This effect can be driven by doctrine since religious minorities often stick more to doctrinal stances than religious majorities. This effect can also be driven by commitment because adherents of religious minorities often are more engaged in their community than members of big churches (Hohenschue et al. 2022). At the same time, however, it is important to note that such a simple categorisation (minority groups, majority groups) overlooks differences within categories. For example, Muslims showed considerably higher agreement with Benevolent Religious Reappraisal (b-value = 0.76) than members of Christian Free Churches (b-value = 0.43) or other religious traditions (b-value = 0.36). The distinction between religious minority and majority groups reaches the limits of its explanatory power at the latest when the different effects of religiosity in the respective groups are examined. None of our hypotheses formulated in this regard could be confirmed.

Nevertheless, it was shown that religiosity could have different influences on religious meaning depending on religious affiliation. This is true not only concerning the effects’ strength but also partly concerning their direction (positive or negative impact). From a theological point of view, this is plausible because the various religions differ in their doctrine. Our results indicate that even in a widely secularised society, doctrinal differences matter to some extent. Therefore, there is a need for further empirical research to understand better how religiosity works in different religious traditions. In particular, the content of the various religious beliefs should receive more attention in future research.

Finally, our findings show how religious meaning-making is affected by the experience of a SARS-CoV‑2 infection. In contrast to the findings of previous studies (Ai et al. 2007; Bjorck and Thurman 2007), our results demonstrate that such an experience does not affect the Punishing God Reappraisal but has a small influence on the Reappraisal of God’s Power. In addition, our findings also show that both the intensity and the direction (positive or negative) of religiosity are not affected by an experience of a SARS-CoV‑2 infection (moderation effect). This result is in line with the thesis of Ai et al. (2007), who claim that the experience of distress is not related to the strength of faith in its effect on religious meaning-making.

Nevertheless, the methodological limitations of our study must also be considered when interpreting its results. The main restriction concerns the sample of the study. As previously described, our sample comprises students and staff at two German universities. This limits the generalizability of the findings in two ways. First, the German context must be taken into account. Germany is a country that has undergone a particular process of secularisation, although there are apparent differences between East and West Germany (Pollack and Pickel 2007). Nevertheless, some countries, for example, in north-western Europe, are much more secularised (Kaufmann et al. 2012). Other countries, on the other hand, do not show such a degree of secularisation or are only at the beginning of such a process (Norris and Inglehart 2007). Therefore, replication studies in other countries are necessary to ensure that the associations we have found are not due to the German context but also have validity in different national and cultural contexts.

Second, students and employees of a university do not represent the population of Germany. Instead, our sample represents a specific selection that, for example, only includes people of a particular age group with a high formal education level. Accordingly, further studies are needed to examine other population groups, such as older people or people with middle and low education levels, to check our results’ validity.

## Appendix

**Table 5:** Documentation of item wording

Variable	Code	Wording
Benevolent God Reappraisal	RCOPE‑1	Saw my situation as part of God’s plan
	RCOPE‑2	Tried to find a lesson from God in the event
	RCOPE‑3	Tried to see how God might be trying to strengthen me in this situation
Punishing God Reappraisal	RCOPE‑4	Wondered what I did for God to punish me
	RCOPE‑5	Decided that God was punishing me for my sins
	RCOPE‑6	Felt punished by God for my lack of devotion
Reappraisal of God’s Power	RCOPE‑7	Questioned the power of God
	RCOPE‑8	Thought that some things are beyond God’s control
	RCOPE‑9	Realized that God cannot answer all of my prayers
Religiosity	CRS‑1	How often do you think about religious issues?
	CRS‑2	To what extent do you believe that God or something divine exists?
	CRS‑3	How often do you take part in religious services?
	CRS‑4	How often do you pray?
	CRS‑5	How often do you experience situations in which you have the feeling that God or something divine intervenes in your life?

## Footnotes

1 For the Catholic church: https://www.katholisch.de/artikel/25004-wilmer-gedanke-von-strafendem-gott-ist-fuerchterlich-und-unchristlich; for the Protestant church: https://www.evangelisch.de/inhalte/168621/10-04-2020/bedford-strohm-will-nichts-mit-einem-strafenden-gott-zu-tun-haben [both retrieved on 10.09.2022].
